# Development of a Multiparametric Voxel-Based Magnetic Resonance Imaging Biomarker for Early Cancer Therapeutic Response Assessment

**DOI:** 10.18383/j.tom.2015.00124

**Published:** 2015-09

**Authors:** Craig J. Galbán, Benjamin Lemasson, Benjamin A. Hoff, Timothy D. Johnson, Pia C. Sundgren, Christina Tsien, Thomas L. Chenevert, Brian D. Ross

**Affiliations:** Departments of 1 Radiology and; 2 Biostatistics, University of Michigan, Ann Arbor, MI;; 3 Faculty of Medicine, Department of Clinical Sciences/Diagnostic Radiology, Lund University, Lund, Sweden; and; 4 Department of Radiation Oncology, Washington University, St. Louis, MO

**Keywords:** treatment response assessment, glioma, imaging biomarker, parametric response map

## Abstract

Quantitative magnetic resonance imaging (MRI)-based biomarkers, which capture physiological and functional tumor processes, were evaluated as imaging surrogates of early tumor response following chemoradiotherapy in glioma patients. A multiparametric extension of a voxel-based analysis, referred as the parametric response map (PRM), was applied to quantitative MRI maps to test the predictive potential of this metric for detecting response. Fifty-six subjects with newly diagnosed high-grade gliomas treated with radiation and concurrent temozolomide were enrolled in a single-site prospective institutional review board-approved MRI study. Apparent diffusion coefficient (ADC) and relative cerebral blood volume (rCBV) maps were acquired before therapy and 3 weeks after therapy was initiated. Multiparametric PRM (mPRM) was applied to both physiological MRI maps and evaluated as an imaging biomarker of patient survival. For comparison, single-biomarker PRMs were also evaluated in this study. The simultaneous analysis of ADC and rCBV by the mPRM approach was found to improve the predictive potential for patient survival over single PRM measures. With an array of quantitative imaging parameters being evaluated as biomarkers of therapeutic response, mPRM shows promise as a new methodology for consolidating physiologically distinct imaging parameters into a single interpretable and quantitative metric.

## Introduction

The prognosis of glioblastoma (GBM) patients remains dismal because with the current standard of treatment patients have an average median survival of only 14 to 16 months (1, 2). Radiation therapy with concomitant temozolomide continues to be the standard of care for treatment of patients with GBM, and new therapies are urgently needed. Improving patient management (and ultimately the outcome) will require more sensitive and optimized imaging outcome metrics that will provide earlier biomarkers of therapeutic response accompanied by improved therapeutic strategies. Determining treatment response and clinical decision-making is currently based on radiologically assessing tumor volume measurements 10 weeks after the initiation of treatment. However, evaluating GBM magnetic resonance imaging scans (MRIs) requires differentiating responding tumors from progressive disease, which can be complicated by alterations in MRI tumor contrast as a result of therapeutically associated disruption of the blood–brain barrier (BBB), a phenomenon termed pseudoprogression that mimics true disease progression (3, 4). Thus, identifying neuroimaging methods that can assess treatment response early as well as distinguishing pseudoprogression from tumor progression would be a major advance in neuroradiology and the clinical management of GBM patients (5). Because changes in imaging tumor volume measurements can be confounded by BBB permeability changes along with inflammatory processes, efforts to improve the accuracy of patient response assessment have resulted in updated standardized clinical response criteria published by the Response Assessment in Neuro-Oncology Working Group (RANO) (4). These new criteria address limitations with the previous response assessment criteria (Macdonald criteria) (6) by advocating the use of additional physiologically sensitive imaging biomarkers for a more accurate tumor response assessment. RANO emphasized that future response criteria will integrate newly advanced MRI techniques such as perfusion imaging (4) after rigorous clinical validation. Thus, new developments will require integrating not only 1 but multiple MRI parameters (anatomical and/or physiological) to simultaneously follow morphological and physiological modifications induced by current and emerging therapies.

Anatomical MRIs, clinical status, and corticosteroid-dependency assessment are the standards for treatment response assessment in GBM patients. Quantitative MRI may provide complementary information related to early changes in tumor pathophysiology following treatment interventions. Quantitative MRI metrics widely under evaluation as metrics of tumor response include relative cerebral blood volume (rCBV) and apparent diffusion coefficient (ADC). Tumor hemodynamics can be assessed and quantified using rCBV metrics obtained through dynamic susceptibility contrast (DSC) MRI exams (7–10). This measure, as well as other MRI-based hemodynamic parameters, has shown promise as a surrogate of clinical outcome for GBM patients treated with chemoradiotherapy as well as with antivascular/antiangiogenic agents (7, 8, 11–13). In addition, diffusion-weighted MRI (DW-MRI) has emerged as a method capable of measuring the random thermal (Brownian) motion of water molecules within tumor tissue and as such is sensitive to therapeutically associated changes in the tumor microenvironment (14–19). A decrease in tumor cellularity resulting from the killing of tumor cells following therapy has been associated with an increase in water diffusivity reflected in the ADC (15). This trend has been observed in preclinical and clinical studies, supporting the notion of DW-MRI as a surrogate imaging biomarker for treatment response assessment in oncology (20–22).

Evaluating the efficacy of quantitative imaging as biomarkers of therapeutic response has relied on obtaining summary statistics (eg, mean and median) over the entire tumor volume. The attractiveness of this analytical approach is the ease of implementation, reduction to a single scalar quantity, and the availability of software to implement such an analysis. Nonetheless, this approach has limitations because statistical measures calculated from quantitative tumor values can become attenuated when tumors exhibit heterogeneities in their response pattern; that is, different parts of the glioma respond differently to treatment (23, 24). To address this issue, a voxel-based method referred to as a parametric response map (PRM) has been developed that overcomes the lack of sensitivity in histogram-based techniques to quantify the evolution of treated tumors using quantitative maps (11, 25). In the context of DW-MRI, PRM is referred to as functional diffusion mapping (fDM) and has been shown to be an independent indicator of overall survival in a cohort of glioma patients (20, 25). The fDM imaging biomarker has also been demonstrated to predict outcomes in bone metastases and breast cancer (26). In 2009, PRM was demonstrated to improve the effectiveness of rCBV (11, 27) in predicting overall survival in glioma patients treated with chemoradiotherapy. In these studies, a larger tumor portion of PRM_rCBV−_ predicted shorter overall survival. More importantly, PRM was described as a general analytical technique that could be applied to multimodal quantitative maps for voxel-based tracking of disease status (11) and progression and has since been applied across a wide variety of imaging parameters (28–30).

This study reports the outcome of a prospective single-center trial of glioma patients that evaluated the ability of multi-PRM (mPRM) to predict overall survival. We incorporated 2 physiologically sensitive quantitative imaging maps (ie, rCBV and ADC) into a single mPRM biomarker response metric. This approach retained detailed spatial information on heterogeneity in the tumor response pattern that was critical for improving the predictive sensitivity of the quantitative maps. This study demonstrates that in our patient cohort incorporating multiple metrics into the mPRM voxel-based analysis approach improved the predictive accuracy of the biomarker over the evaluation of a single biomarker.

## Methodology

### Patients

Patients with pathologically proven high-grade gliomas were enrolled on a protocol of intratreatment MRI as part of a single-site prospective trial. Informed consent was obtained, and the use of images and medical records was approved by the University of Michigan Institutional Review Board. Only patients with contrast-enhancing tumors of 4 cm^3^ or greater were included in this study. Fifty-six patients were evaluated before therapy and 3 weeks after the initiation of chemoradiotherapy (Table 1), which consisted of concomitant and adjuvant temozolomide with radiation. Part of this patient cohort had been used previously in published work that investigated the prognostic value of PRM applied to the individual parameters ADC and rCBV and a composite model of PRM applied to these 2 parameters (11, 20, 31).

**Table 1.** Clinical Characteristics

Variable	All Patients (n = 56)		Responder (n = 13)		Intermediate (n = 20)		Nonresponder (n = 23)		*P* Value
	No.	%	No.	%	No.	%	No.	%	
Age (y)	55 (15)		50 (15)		49 (12)		63 (13)		**.002**
Sex									
Male	24	43	6	46	10	50	8	35	.58
Female	32	57	7	54	10	50	15	65	
Pathology (grade)									
3	10	18	4	31	2	10	4	17	.32
4	46	82	9	69	18	90	19	83	
Karnofsky performance test									
<70	43	77	3	23	2	10	8	35	.14
≥70	13	23	10	77	18	90	15	65	
Location									
Frontal/temporal	35	63	9	69	12	60	14	61	.85
Other	21	37	4	31	8	40	9	39	
Tumor volume (mL)	37 (27)		43 (22)		22 (15)		47 (33)		**.01**
Radiological response*									
Stable disease/partial response	24	52	10	91	8	53	6	30	**.02**
Progressive disease	20	44	1	9	6	40	13	65	
Surgery									
Biopsy	21	38	2	15	5	25	14	61	**.04**
Subtotal	23	41	8	62	9	45	6	26	
Near guided tissue regeneration	12	21	3	23	6	30	3	13	
Radiation therapy (Gy)	68 (9)		70 (8)		68 (9)		66 (9)		.5
Chemotherapy*									
Any	54	96	13	100	20	100	21	91	.16
Temozolomide	52	93	13	100	20	100	19	86	.06
Temozolomide + radiotherapy	31	55	8	62	13	65	10	44	.32

Bold *P* values indicate statistical significance. The asterisk indicates omitted data for radiological response (10 missing and 2 not available) and chemotherapy (1 missing for “Any”). Likelihood ratio was performed for sex, grade, Karnofsky performance test, location of surgery, and chemotherapy. An ANOVA with a Bonferonni post hoc test was used for age, tumor volume, and radiation therapy dose.

Radiotherapy was delivered over 6 weeks using standard techniques with a 2.0 to 2.5-cm margin on either the enhancing region on gadolinium (Gd)-enhanced scans or the abnormal signal on T2-weighted scans to 46 to 50 Gy, with the central gross tumor treated to a final median dose of 70 Gy. Chemotherapy was commonly administered in both groups depending upon clinical circumstances (Table 1). No patient received antiangiogenic therapy during their primary treatment, although some received bevacizumab (Avastin; Genentech) at the time of progression.

### MRIs

MRIs were performed 1 week before therapy and 3 weeks after therapy had begun. DW- and dynamic susceptibility contrast (DSC)-MRI and standard MRI (fluid attenuation inversion recovery, T2-weighted, and Gd-enhanced T1-weighted MRI) were conducted on either a 1.5 T Signa (General Electric Med*i*cal Systems) or 3 T Achieva (Philips Medical Systems) system. In this study, repeat scans were always acquired on the same MRI scanner as the baseline scan. Radiologic response at 10 weeks was based on changes in tumor volume on T1-weighted contrast-enhanced MRIs and steroid doses and was classified as complete response, partial response, stable disease, and progressive disease (6). Steroid doses were recorded before each scan, weekly during radiotherapy, and at each follow-up (32).

DW-MRIs were acquired using a single-shot, spin-echo, diffusion-sensitized, echo planar imaging acquisition sequence. Scans acquired on the 1.5 T system were obtained from 24 6-mm axial-oblique sections using a 22-cm field of view (FOV) and 128 × 128 matrix with *b* factors of 0 and 1000 s/mm^2^ along 3 orthogonal directions (repetition time [TR] = 1000 ms; echo time [TE] = 71-100 ms; number of averages [NS] = 1). Images obtained using the 3 T MRI scanner acquired at least 28 4-mm axial-oblique sections through the brain using a 24-cm FOV and 128 × 128 matrix (TR = 2636 ms; TE = 46 ms; NS = 1 for a *b* value of 0 and NS = 2 for a *b* value of 1000 s/mm^2^). Parallel imaging (sensitivity encoding factor = 3) was used on the 3 T scanner to reduce spatial distortion. The diffusion-weighted images for the 3 orthogonal directions were used to calculate ADC maps for all patients (20).

To obtain DSC-MRI data, a gradient echo planar imaging pulse sequence was used with the following acquisition parameters: TR = 1.5 to 2 s; TE = 50 to 60 ms; FOV = 22 cm; matrix = 128 × 128; flip angle = 60°; 4-6-mm thickness; 14-20 slices; 0-mm gap. Gd-DTPA (Bayer HealthCare Pharmaceuticals) was injected intravenously with a dose of 0.05 to 0.1 mL/kg as a bolus using a power injector at a rate of 2 mL/s and followed immediately by 15 cc of saline flush at the same rate. A Gd-enhanced T1-weighted image was then acquired. All CBV maps were computed from DSC images as previously described (33). To mitigate the effects from leakage, a preinjection of contrast agent before a second bolus was given during the dynamic T2* imaging (ie, DSC-MRI). In addition, a sufficiently long TR was employed to reduce T1 weighting. To assess differences in tumor blood volume during chemoradiotherapy and among patients, all CBV maps were normalized to CBV values in white matter regions that were contralateral to the tumor to generate rCBV maps. (For simplicity in notation, relative blood volumes for both brain and tumor have been denoted by the abbreviation rCBV throughout this article.) White matter regions of interest that were used for normalization were contralateral to the tumor and regions that received an accumulated dose < 30 Gy and avoided regions of partial volume averaging or regions with susceptibility artifacts.

### Postprocessing Images

All image data were registered to pretreatment Gd-enhanced T1-weighted images using mutual information as an objective function and the Nelder–Mead simplex as an optimizer (34). Both differently and similarly weighted serial MRIs for the same patient were registered assuming a rigid-body geometric relationship (ie, rotate and translate). After registration, brain tumors were manually contoured by a neuroradiologist over the contrast-enhancing regions of the tumor on Gd-enhanced T1-weighted images.

The PRM of any single parameter (PRM_X_, where X denotes any parametric map such as ADC and rCBV) was determined by first calculating the difference between X (ΔX = mid-X − baseline X) for each voxel within the tumor before and 3 weeks after the initiation of treatment. Voxels that yielded a ΔX value greater than a predetermined threshold were designated red (ie, ΔrCBV > 1.2; ΔADC > 55; PRM_X+_). Blue voxels represented volumes whose parameter value decreased by more than the threshold (ie, ΔrCBV <−1.2; ΔADC <−55; PRM_X−_), and green voxels represented voxels within the tumor that were unchanged (ie, |ΔrCBV| < 1.2; |ΔADC| < 55; PRM_X0_). Thresholds were set to 1.2 and 55 for rCBV and ADC, respectively, as determined from previously published work (11, 20, 25). In brief, healthy contralateral brain tissue from registered parameter maps was contoured to generate voxels with paired parameter values at baseline and 3 weeks after treatment was initiated. A linear regression was applied to the data, and the 95% confidence interval (CI) of the fit was used as the threshold for each of the parameters. Thresholds were determined using a subset of patients from this cohort as described in previously published studies (11, 20, 25).

**Figure 1.**
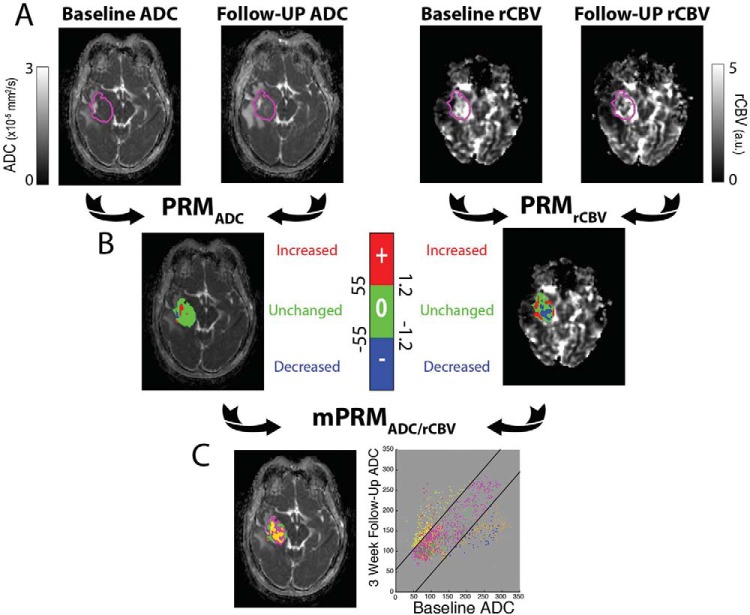
Schematic of the multiparametric PRM technique. (A) Parametric maps of ADC from DW-MRI (left) and rCBV from DSC-MRI (right) acquired before and midway through therapy. (B) The PRM approach was applied to the individually ADC and rCBVs, resulting in 3 classifications each. PRM results are presented as a 3-color overlay that represents regions in which tumor parameter values (ie, ADC or rCBV), based on predetermined thresholds, are unchanged (green voxels), significantly increased (red voxels), or significantly decreased (blue voxels). (C) The individual PRMs are combined, resulting in 9 classifications (2 parameters and 3 classes, resulting in 3^2^ = 9 classes for mPRM). Analogous to mPRM, results are presented as a visual map in which individual voxels are colored by classification as well as presented within a scatter plot. Global measures are presented as relative tumor volumes and calculated as the sum of all voxels in a class normalized to the tumor volume.

Multiparametric response mapping was applied to ADC and rCBV to generate analytical indices sensitive to changes in both quantitative maps (Figure 1). After image registration as previously described, all serial ADC and rCBV maps shared the same spatial geometric space, with each voxel having temporal pairs for each biomarker: baseline and midtreatment values of ADC and rCBV. As described previously, PRM applied to a single biomarker results in 3 classifications; when applied to 2 biomarkers voxels it is separated into 9 classifications (3 classifications per biomarker and 2 biomarkers [3^2^] equal 9 classifications.) The notation used to indicate the mPRM classifications is analogous to PRM for a single biomarker (Figure 1). For example, the volume fraction of tumor characterized as having red on the PRM_ADC_ map (PRM_ADC+_) and blue on the PRM_rCBV_ map (PRM_rCBV−_) is named mPRM_ADC+/rCBV−_.

### Statistical Analysis

The 3 classifications for PRM_ADC_ and PRM_rCBV_ and all 9 classifications generated by mPRM_ADC/rCBV_ were assessed for predicting 1-year survival using a receiver operating characteristic (ROC) analysis. The patient population was stratified for each significant metric per PRM model (ie, single and multiparametric) based on optimal cutoffs that maximize sensitivity and specificity in the ROC analysis. Significant single PRM indices were used to generate a 3-tier (ie, composite) PRM model using the same procedure as described in previously published work (31). In brief, PRM_ADC_ and PRM_rCBV_ indices that resulted in a significant prediction of 1-year survival after ROC analysis were combined to stratify the patient population into 3 categorical therapeutic assessments: responders, nonresponders, and intermediate. Patients were designated nonresponders when both PRM indices predicted nonresponding, whereas patients were designated responders when both PRM indices predicted responding. For cases in which PRM indices disagreed, patients were designated intermediate. Because there are 9 metrics generated by the mPRM analysis, multiple metrics may significantly stratify the patient population based on 1-year survival. In this case, the same 3-tier procedure in combining patient stratification by PRM_ADC_ and PRM_rCBV_ was applied to the significant mPRM indices. Assessing overall survival for all PRM and mPRM models was determined using a Kaplan–Meier analysis with a log-rank test. A multivariate Cox regression with forward entry was used to compare the 3-tier mPRM model to the single-parameter PRM, 3-tier PRM, and individual mPRM models. A secondary analysis was conducted to assess differences in clinical characteristics among outcome groups defined by the 3-tier mPRM model using either a univariate ANOVA with a Bonferroni post hoc test to control for multiple comparisons or a likelihood ratio test. All statistical computations were conducted with a statistical software package (SPSS Software Products), and results were declared statistically significant at the 2-sided 5% comparison-wise significance level (*P* < .05).

## Results

### Patient Characteristics

A total of 57 patients with high-grade glioma was included in this prospective study (Table 1). The median survival for the population, as determined by the Kaplan–Meier analysis, was 12.8 months (95% CI = 7.2, 18.4), with 50% of the patient population realizing 1-year survival. Patients who died before 1 year had a median survival of 7 months (95% CI = 5.4, 8.6), whereas those whose survival was beyond 1 year had a median survival of 35.1 months (95% CI not available).

**Figure 2.**
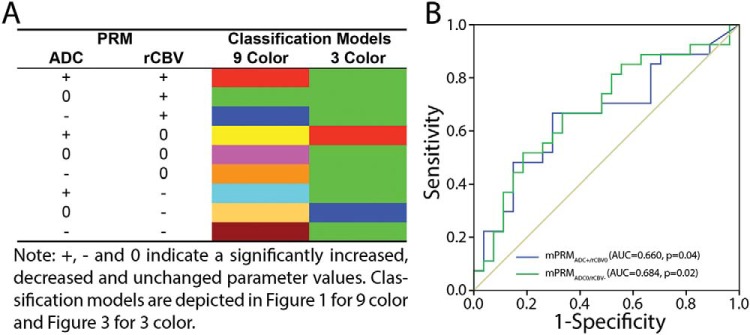
Assessment of predictive mPRM indices. (A) For ease of visualization, the 9 classes generated from the individual PRMs, in which plus (+), minus (−), and 0 represent voxel values that increase, decrease, or remain unchanged, respectively (color code demonstrated in Figure 1), were simplified to a 3-classification scheme. Voxels were color-coded as red for mPRM_ADC+/rCBV0_, blue for mPRM_ADC0/rCBV−_, and green for the remaining 7 classifications. (B) Predictive mPRM indices were identified by assessing the potential of the 9 indices to predict 1-year survival using an ROC analysis. Significant ROC stratification was observed in 2 of the 9 classes: mPRM_ADC+/rCBV0_ and mPRM_ADC0/rCBV−_. These results were used to determine the cutoffs that maximize both sensitivity and specificity for each metric. Cutoffs for response stratification were determined to have relative volumes (RVs) of 2.6% for mPRM_ADC+/rCBV0_ (responders: RV ≥ 2.6%; nonresponders: RV < 2.6%) and 4.8% for mPRM_ADC0/rCBV−_ (responders: RV < 4.8%; nonresponders: RV ≥ 4.8%).

mPRM was analyzed by first generating PRM results for individual metrics by spatially aligning temporally paired ADC and rCBV maps to a single geometric frame (Figure 1A) and then classifying voxels based on previously published thresholds (Figure 1B) (11, 20). Merging the individual PRM data to a single new mPRM resulted in 9 classifications based on changes of both ADC and rCBV at the voxel level (Figures 1C and 2A). Key mPRM classifications were identified using an ROC analysis to assess the potential of each of the 9 classifications for predicting 1-year survival.

In all, 2 of the 9 classifications were identified as predictive of 1-year survival (Figure 2B). The mPRM indices with significantly increasing ADC values and unchanged rCBV (mPRM_ADC+/rCBV0_) values had an area under the curve (AUC) of 0.66 (*P* = .04), whereas mPRM indices with unchanged ADC and significantly decreasing rCBV values (mPRM_ADC0/rCBV−_) had an AUC of 0.684 (*P* = .02). The remaining 7 indices had an AUC near random, with *P* > .4. To simplify the graphical representation on a 3-dimensional image, all voxels classified as mPRM_ADC+/rCBV0_ were color-coded red, those classified as mPRM_ADC0/rCBV−_ were blue, and the remaining voxels were green (Figure 2A). The optimal cutoffs for patient stratification for mPRM_ADC+/rCBV0_ and mPRM_ADC0/rCBV−_ were found to be 2.6% and 4.8% of the tumor volume, respectively. Near-significant results from ROC analyses were observed for PRM_ADC+_ (AUC = 0.651, *P* = .057, cutoff = 4.8%) and PRM_rCBV−_ (AUC = 0.650, *P* = .058, cutoff = 5.5%).

**Figure 3.**
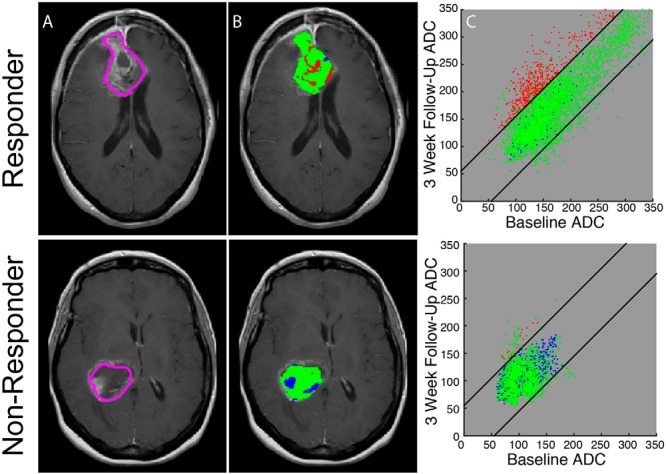
Representative slices from patients identified as responders and nonresponders by mPRM. (A) Presented are T1-weighted post-Gd axial slices with a tumor contour generated from intersecting pretreatment and 3-weeks-of-treatment contours and (B) mPRM overlays with (C) a corresponding mPRM scatter plot for patients identified by mPRM as responder (top) or nonresponder (bottom). The responder was identified as having relative tumor volumes of 10% and 2% for mPRM_ADC+/rCBV0_ and mPRM_ADC0/rCBV−_, respectively. In contrast, the nonresponder had relative tumor volumes of 0% and 14% for mPRM_ADC+/rCBV0_ and mPRM_ADC0/rCBV−_, respectively. Both responders and nonresponders were diagnosed as having stable disease by the Macdonald criteria yet had overall survivals of 64 and 7.1 months, respectively. Scatter plot axes are presented as pretherapy ADC (ie, baseline) for the *x*-axis and 3-week follow-up ADC for the *y*-axis. Color coding is based on the 3-color mPRM classification model shown in Figure 2B.

Figure 3 presents representative axial mPRM slices and corresponding scatter plots from 2 patients in which both indices of mPRM identified each patient as a responder or nonresponder 3 weeks into therapy. Figure 2A shows the color coding for the 3-color model. The responder was found to have an mPRM_ADC+/rCBV0_ at 10% of the tumor volume, with 2% of the tumor classified as mPRM_ADC0/rCBV−_. In contrast, the tumor volume from the nonresponder consisted of less than 1% of mPRM_ADC+/rCBV0_, yet 14% consisted of mPRM_ADC0/rCBV−_. Although both patients were diagnosed as having stable disease by the Macdonald criteria, the overall survivals were quite different, with the responder and nonresponder having survival times of 64 and 7 months, respectively.

**Figure 4.**
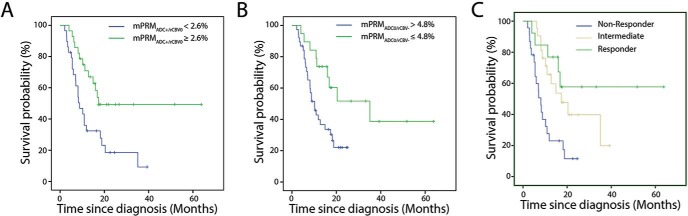
Survival analysis of mPRM biomarkers. Kaplan–Meier survival plots for overall survival are presented for mPRM_ADC+/rCBV0_ (A) and mPRM_ADC0/rCBV−_ (B), where subjects are stratified based on cutoffs determined by ROC analysis (see Figure 2). (C) Overall survival plot for the 3-tier mPRM model. This model separates subjects into 3 groups: responders (mPRM_ADC+/rCBV0_ ≥ 2.6%; mPRM_ADC0/rCBV−_ ≤ 4.8%), nonresponders (mPRM_ADC+/rCBV0_ < 2.6%; mPRM_ADC0/rCBV−_ > 4.8%), and intermediate (remaining combinations).

For an imaging biomarker to be clinically feasible for treatment management, it must be demonstrated as a surrogate marker of overall survival. Figure 4 shows the Kaplan–Meier survival plots for the mPRM indices as well as the combined mPRM model. All analyses produced significant results. As observed in Figure 4A, mPRM_ADC+/rCBV0_ generated a *P* = .01, whereas those identified as nonresponders (n = 29) had a median survival of 8.8 months (95% CI = 5.6, 12.0), and responders (n = 27) had a median survival of 17.4 months (95% CI not available). Similar results were observed for mPRM_ADC0/rCBV−_, with a *P* = .01 and median survivals for nonresponders (n = 37) and responders (n = 19) as 10 months (95% CI = 7.5, 13) and 35 months (95% CI = 10, 60), respectively.

Combining the 2 mPRM indices into a 3-tier model improved the potential of the mPRM to predict overall survival (Figure 4C; *P* = .002). Median survivals of the 3 groups were 8.1 months (95% CI = 6.1, 10) for nonresponders, 17 months (95% CI = 8.5, 26) for intermediate, and unattained for responders. A pairwise comparison of the individual pools generated significant results only when comparing nonresponders to either intermediate (*P* = .007) or responders (*P* = .005). No significant differences were observed between intermediate and responders (*P* = .2). When comparing the model to the patient clinical metrics (Table 1), the combined mPRM model was found to significantly vary only for patient age (*P* = .002), initial tumor volume (*P* = .01), radiological response (*P* = .02), and surgical extent (*P* = .04). In short, patients identified as nonresponders were found to be older, had larger initial tumors, underwent biopsies and, as expected, were diagnosed as having progressive disease by radiological response.

Including the intermediate group demonstrated improved performance in the 3-tier model for predicting overall survival beyond what was observed in the single mPRM indices as well as the single-parameter PRM and composite PRM models. Further confirmation was established using a Cox regression analysis with forward entry to assess in a multivariate statistical model which of the single mPRM or PRM schemes provided a better fit to the survival data than what was observed by the 3-tier mPRM model. The Cox regression showed that no further improvement was obtained beyond the inclusion of the 3-tier mPRM in the statistical model (*P* = .003). Controlling for patient age, a known predictor of outcome, did not alter the statistical model results.

## Discussion

Because of limited treatment efficacy and radiological phenomena such as pseudoprogression, accurately assessing the therapeutic response of gliomas remains a challenge. The updated response assessment criteria for high-grade gliomas reaffirmed the use of anatomical MRI for assessing tumor response to treatments (4). These recommendations suggest evaluating tumor evolution over time based on structural changes observed on both T1-weighted post-Gd and fluid attenuation inversion recovery images 10 to 12 weeks after the start of treatment. Recent studies have shown that morphological changes occur after physiological modifications that may be captured by quantitative MRI techniques (11, 25). The goal of this study was to evaluate a voxel-based approach that generates a response biomarker from 2 physiologically sensitive parameters capable of assessing patient survival after treatment. In this study, mPRM was demonstrated using ADC and rCBV maps and evaluated for how well it predicted overall survival of glioma patients accrued as part of a single-site prospective imaging trial.

Voxel-tracking techniques for assessing responses that demonstrated the value of separating image voxels based on changes in the voxel parametric value from pre- to midtherapy was first introduced in 2005 (25). This approach strongly correlated with a radiological response beyond what was observed using whole-volume tumor summary statistics derived from a histogram analysis (ie, mean change). The voxel-based technique PRM has been shown to be an independent indicator of overall survival as early as 3 weeks after treatment is initiated when applied to either ADC or rCBV and the composite of both (11, 20, 31). As previously reported, PRM_ADC+_, PRM_rCBV−_, and the composite PRM model were also found to significantly stratify the patient population (*P* = .012, .033, and .005, respectively). Although mPRM is analogous to previously published work, the strength of this methodology versus its predecessors is the retention of spatial information that is lost in the composite of PRM_ADC+_ and PRM_rCBV−_ (31). The mPRM technique is a truly voxel-based technique—not a pseudorepresentation of individual PRM analyses of ADC and rCBV. Only 2 of the 9 possible metrics derived from mPRM, mPRM_ADC+/rCBV0_, and mPRM_ADC0/rCBV−_ were found to be highly predictive of survival after chemoradiotherapy. When combined into a 3-tier model, the potential for predicting overall survival was found to improve over the individual mPRM biomarkers. This highlights the importance of utilizing methods that integrate multiple parameters into a single response map for treatment assessment that can retain spatial information.

Although DW-MRI has become a routine sequence used in most neurological exams, precautions must be taken when acquiring DSC-MRIs to reduce errors in the rCBV measurements. This study implemented all recommendations by Paulson et al. (35) for reducing the impact of the BBB leakage and the T1-weighting effect on CBV measurements. In short, the following protocols were employed: (1) a preinjection of contrast agent before a second bolus (used for the DSC-MRI experiment) to decrease the BBB leakage, (2) a long TR (1.5 to 2 s) to further reduce the T1-weighting effect, and (3) the measurement and use of the arterial input function for each DSC experiment.

The mPRM technique is an extension of the PRM method described in previously published work and as such raises the same issues: changes in the volume of interest size over time and registration. As such, no significant changes of tumors sizes were observed before or 3 weeks after the initiation of treatment in this patient cohort. Moreover, variations observed were below interobserver variability as previously reported for manually tracing brain tumors (36). Problems linked to registration have been extensively discussed in several publications (11, 37, 38). These studies indicate that image-processing approaches can be used effectively to coregister brain MRI data, and registration techniques that apply deformable registration algorithms are also widely available and provide ample flexibility for using mPRM in a wide variety of clinical studies. Because of the limited patient cohort used in this study, the patient characteristics presented in Table 1 were not controlled for in the Cox regression statistical model when evaluating the different PRM indices. Nevertheless, the mPRM technique can potentially provide a new imaging methodology for assessing treatment response using multiple parametric maps in brain tumor patients. The efficacy of this technique will have to be verified in larger prospective clinical trials, and trials that evaluate newly targeted drugs such as antiangiogenics will be need to be conducted to confirm these findings.

## Summary

In this study, we demonstrated in a single-site prospective clinical study an early imaging biomarker to distinguish responding from nonresponding glioma patients to standard-of-care chemoradiotherapy. When evaluated by mPRM and rCBV, ADC metrics, individually known to predict treatment failure, were remarkably found to be spatially delocalized, possibly as a result of different tumor response mechanisms, that is, cell kill as quantified by ADC changes and hypoxia as quantified by a reduction in rCBV values. Merging these parametric biomarkers into a single unique and spatially resolved metric provided additional sensitivity over individual metrics. The mPRM technique shows significant promise as an early and robust imaging biomarker of clinical outcome in patients diagnosed with high-grade gliomas. The application of mPRM could be further extended to evaluate a wide variety of tumor types and other disease processes. Moreover, mPRM could be adapted to include a combination of alternative MRI-based metrics, including vascular permeability, blood flow, and extravascular leakage as well as metrics from other modalities, including, for example, positron emission and computerized tomography. Overall, mPRM is a unique and generalizable imaging biomarker capable of being applied for quantifying dynamic and spatially varying alterations in pathologically relevant disease processes and effects of treatment over time.
